# When habitat matters: Habitat preferences can modulate co-occurrence patterns of similar sympatric species

**DOI:** 10.1371/journal.pone.0179489

**Published:** 2017-07-26

**Authors:** César Augusto Estevo, Mariana Baldy Nagy-Reis, James D. Nichols

**Affiliations:** 1 Independent Researcher, Paulínia, São Paulo, Brazil; 2 Department of Animal Biology, University of Campinas, Campinas, São Paulo, Brazil; 3 Department of Wildlife Ecology and Conservation, University of Florida, Gainesville, Florida, United States of America; University of Alberta, CANADA

## Abstract

Disentangling the role of competition in regulating the distribution of sympatric species can be difficult because species can have different habitat preferences or time use that introduce non-random patterns that are not related to interspecific interactions. We adopted a multi-step approach to systematically incorporate habitat preferences while investigating the co-occurrence of two presumed competitors, morphologically similar, and closely related ground-dwelling birds: the brown tinamou (*Crypturellus obsoletus*) and the tataupa tinamou (*C*. *tataupa*). First, we used single-species occupancy models to identify the main landscape characteristics affecting site occupancy, while accounting for detection probability. We then used these factors to control for the effect of habitat while investigating species co-occurrence. In addition, we investigated species present-time partitioning by measuring the degree of overlap in their activity time. Both species were strictly diurnal and their activity time highly overlapped (i.e., the species are not present-time partitioning). The distribution of the two species varied across the landscape, and they seemed to occupy opposite portions of the study area, but co-occurrence models and species interaction factors suggested that the tinamous have independent occupancy and detection. In addition, co-occurrence models that accounted for habitat performed better than models without habitat covariates. The observed co-occurrence pattern is more likely related to habitat preferences, wherein species segregated by elevation. These results provide evidence that habitat characteristics can play a bigger role than interspecific interactions in regulating co-existence of some species. Therefore, exploring habitat preferences while analyzing co-occurrence patterns is essential, in addition to being a feasible approach to achieve more accurate estimation of parameters reflecting species interactions. Occupancy models can be a valuable tool in such modeling.

## Introduction

Interspecific competitive interactions can shape species distributions, abundances, dynamics, co-occurrence patterns, and behavior [1, 2, 3]. Sympatric species may face exclusion, avoidance [4], or even extinction [5] because of competitive interactions, which are usually stronger between species that share similar ecological niches [6]. Therefore, mechanisms that decrease niche overlap usually help similar species persist in sympatry [1]. Such mechanisms include differences in habitat selection and dietary preferences, as well as temporal segregation [1, 7, 8, 9]. In cases where asymmetric competition against congeners is evident, dominant species may adopt an aggressive behavior to displace subordinate species to less preferred habitats [10, 11, 12], or to less preferred times of day [9].

However, disentangling the potential role of competition in regulating species occurrence can be difficult, especially considering that species can have different habitat preferences, which introduce non-random patterns of species occurrence that are not related to interspecific interactions [13]. In addition, not dealing with false absences of species (i.e., non-detections) also can result in erroneous interpretations about species interaction, because these can result in biased estimation of species co-occurrence and interaction frequency [4, 14, 15]. Therefore, a framework that permits analysis of species interaction while dealing with imperfect detection and incorporating habitat characteristics directly into the modeling is more likely to result in accurate estimates of species co-occurrence [4, 14, 16]. Co-occurrence models allow one to estimate the occurrence patterns of multiple species at a single site while explicitly fitting habitat covariates and investigating changes in occupancy and detection of one species in response to the presence of another [3, 4, 13, 14, 17]. For example, mouse lemurs avoid areas where the occupancy of predator is high [18]. In rails, black rails detection probability is unaffected by the detection of Virginia rails, but their occupancy is positively associated [17]. These recent studies highlight that analyses directed at hypotheses of co-occurring species need to deal adequately with imperfect and variable detection probabilities for both species.

In the Tropics, many species of birds live in sympatry within the same area [7, 19, 20], but the mechanisms behind these patterns are still unknown. Here, we investigate the co-occurrence of two presumed competitors, morphologically similar, and closely related bird species while systematically incorporating habitat preferences and imperfect detection into the analysis. More specifically, we used single- and two-species occupancy models to investigate the effects of habitat and interspecific interactions on the co-occurrence patterns of brown tinamou (*Crypturellus obsoletus*) and the tataupa tinamou (*C*. *tataupa*).

Tinamous (Aves: Tinamidae) are endemic to the Neotropical region of South and Central America, and are among the oldest families in the New World. The species have consistent and similar body type, with rounded and compact shape, short tail and wings, stout feet and legs, and cryptic coloration [21, 22]. Tinamous are ground-dwelling birds specialized in exploring only lower levels of the forest strata, and generally defend small territories by adopting aggressive behaviors against each other [21]. Their diet appears to be similar among congeners and consists mostly of fruits, leaves and seeds [21]. Tinamous have occupied a variety of environments, from cloud forests to arid and semi-arid steppes [21, 22], and are found in sympatry [21, 22, 23, 24]. Natural history studies have suggested that competition could be the leading factor influencing tinamou distribution [21, 22], but such studies often lack robust inference methods. Moreover, little is known about most tinamou species, especially forest species, including brown tinamou and tataupa tinamou, for which basic research is still needed [25]. Given the close phylogeny, morphological similarity, and territorial behavior of tinamous [21, 22, 26], they seem to be ideal candidates to investigate patterns of co-occurrence among sympatric similar birds.

We investigated the following competing hypotheses about the co-occurrence pattern of the brown tinamou and the tataupa tinamou. 1) They exhibit habitat partitioning–we expect that their occurrence would vary according to habitat characteristics, and that they would have different habitat preferences. Under this hypothesis, we predicted that one species would not affect the probability of occurrence or detection of the other, after accounting for these habitat differences, which encompass the main habitat features of the study area. 2) They co-occur less frequently than expected by chance because of direct and ongoing competition (either by interference or by exploitative competition)–we expect them to co-occur less frequently than expected under a hypothesis of independence. 3) They are simultaneously influenced by both habitat selection and direct competition–we expect the distribution of the two tinamous to be simultaneously influenced by habitat and competitive interactions. 4) They use similar habitats and co-occur but have time partitioning as a mechanism to limit behavioral interactions–we expect that the two specis would present low overlap in their time activity. Moreover, the more dominant species would be more active in the more favorable period of the day (i.e., early morning and late afternoon; [27]), while the presumably subordinate species would be more active in alternative hours of day (e.g., afternoon).

## Methods

### Study area

**Fig 1:**
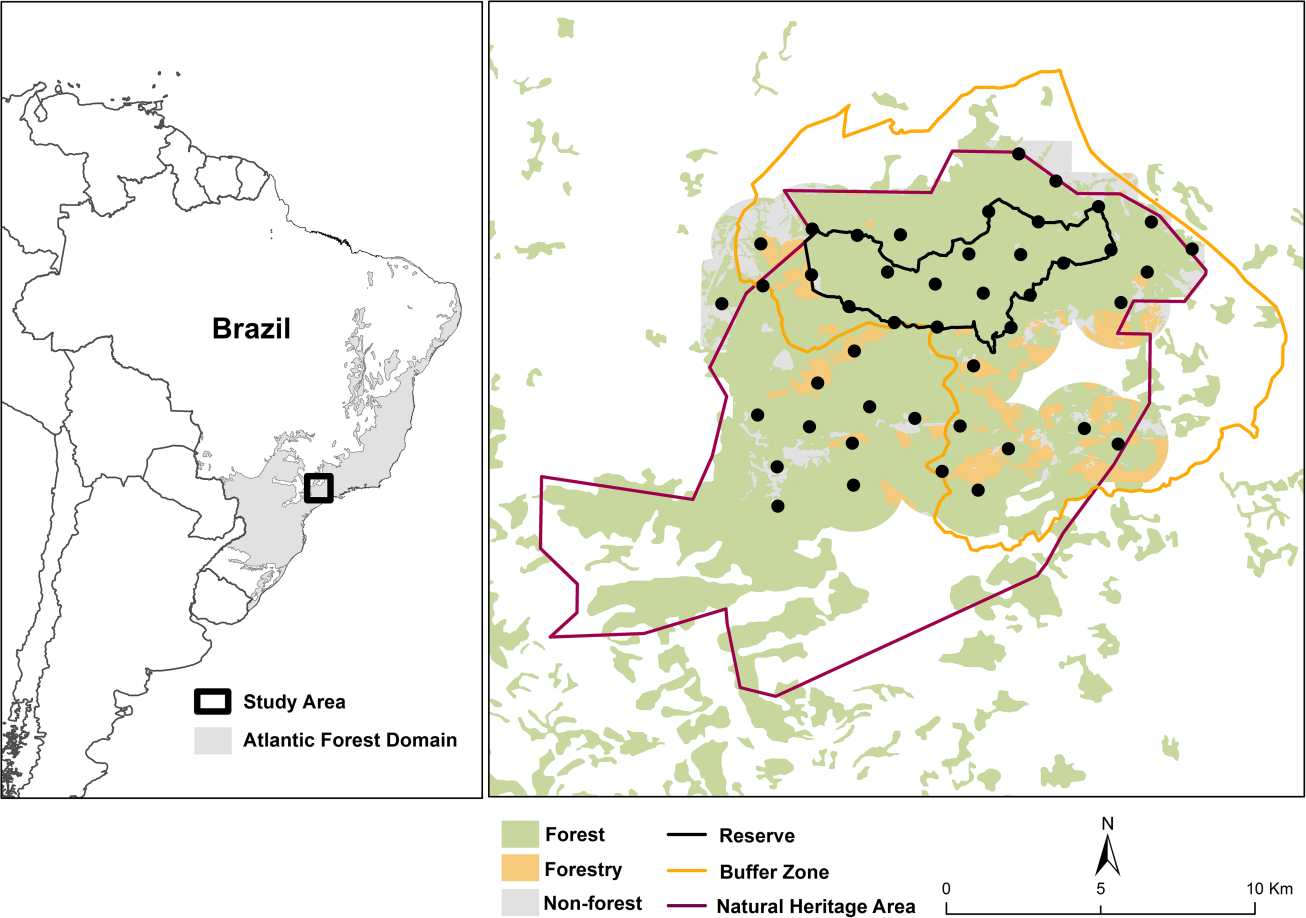
Study area, and location and distribution of sampling sites at Serra do Japi (Brazil) where the brown tinamou (*Crypturellus obsoletus*) and tataupa tinamou (*C*. *tataupa*) were sampled using camera traps. Different vegetation cover types and protection status are also indicated. Map adapted from [31].

We conducted this study at Serra do Japi (coordinates 47°03'40"W to 46°52'20"W and 23°22'30"S to 23°11'35"S, Fig 1), a Natural Heritage Area (35,000 ha) located in southeast Brazil. The area represents one of the few large remnants of Atlantic Forest. This is a global hotspot for biodiversity conservation [28] and is part of the UNESCO’S Atlantic Forest Biosphere Reserve network. Located within the Natural Heritage Area is the Biological Municipal Reserve (REBIO), which follows buffering concepts similar to those proposed by UNESCO [29], with a core area (REBIO; 2071 ha) surrounded by a buffer zone (11, 946 ha) (Fig 1). The REBIO represents the highest protection status in the area, wherein only research and education are allowed. The area is characterized by semi-deciduous mesophile forest with mountainous terrain. The climate is temperate humid, with mean temperature of 19.7°C and mean annual rainfall of 1422 mm, with a wet and warm season from October to March, and a dry and cold season from April to September [30].

### Data collection

We collected data on the brown tinamou and tataupa tinamou with passive infrared non-baited camera traps (Bushnell Trophy Cam; fixed about 20 cm above ground), a valid method to sample ground-dwelling birds [32, 33], including tinamous [32, 34, 35,36]. We performed a survey in the dry season (April 2013 to September 2013), and another in the wet season (October 2013 to March 2014), with a total of 5198 trap days. We used 45 sampling sites uniformly distributed in a virtual regular grid with approx. 1.5 km spacing (177 ha sampling area per sampling site), and placed one camera trap near the center of each cell (see [31] for a representative trap and study design). The spacing between sites ensures that sampling sites are spatially independent because tinamous have small home ranges (approx. 20 ha) and conforms to the TEAM Terrestrial Vertebrate Monitoring Protocol Implementation Manual [37].

### Covariates

We obtained mapping and covariates from previous studies that we have conducted at the same study area, and used a similar multi-scale approach (see [31, 38] for details on mapping and covariate aquisition). Land use and hydrographic density were mapped with Quantum GIS software [39] using high resolution satellite image interpretation at a 1:5000 scale, cartographic maps at a 1:10000 scale (Secretariat of Economy and Planning, São Paulo State Government), and extensive field verification. Covariates were obtained in different spatial scales (i.e., concentric circles—buffers) of 200-, 300-, 500- and 1000-m radius around each sampling site. The smaller buffers (which cover 12 ha and 28 ha, respectively) are likely to represent the tinamous’ home range scale (approx. 20 ha, based on the home range of *Crypturellus variegatus* [40], and *C*. *boucardi* [41]), whereas the larger buffers (78 ha and 314 ha, respectively) can be regarded as landscape scales.

The covariates used to model occupancy were: mean elevation, hydrographic density, percentage of high-quality vegetation cover, and weighted distance to the Biological Municipal Reserve (REBIO) border [31]. Elevation data was obtained from digital elevation models (DEM) from Topodata Geomorphic database of Brazil [42]. Hydrographic density was estimated using the Kernel density function in ArcGIS software [43]. The percentage of mid and late forest succession were considered as indicators of high-quality vegetation cover, and were calculated with Geographical Resources Analysis Support System (GRASS; [44]). Furthermore, the distance of each sampling site to the nearest REBIO border was measured, giving negative distances to sites within the REBIO or positive distances otherwise (i.e., the center of the reserve received the smallest values). Then, the distance of each sampling site was multiplied by the protection status weight of the area where the sampling site was located (REBIO = 1; REBIO’s buffer zone = 2; within the Natural Heritage Area but outside these two areas = 3; outside these three areas = 4), obtaining the ‘weighted distance to REBIO border’.

Our purpose in measuring these covariates was to control for the effect of habitat over species’ occupancy and co-occurrence. We predicted that high-quality vegetation would be the main environmental covariate to influence tinamous occupancy, as both seem to prefer forested habitats [22]. To a minor degree, the proximity to the REBIO would positively influence their occupancy, as both tinamous are game species and their abundance may decrease in hunted areas [45,46]. We expected elevation to have an effect if the birds were segregating along an elevational gradient due to the mountainous terrain in our study area (similarly to [14]). We predicted that hydrographic density would have a positive effect on occupancy of both species, as this variable reflects water availability, and tinamous drink water regularly and bath in streams [21, 22]. We normalized all covariates and used only covariates with low correlation (r < 0.50), as assessed with a Spearman’s correlation matrix (Table A in S1 File).

To model detection probability, we considered mean temperature, total precipitation, season (dry and wet), and terrain slope at the sampling site. All climate variables were obtained from the Integrated Center of Agrometeorology Information [47], and terrain slope from digital elevation models (DEM) from the Topodata Geomorphic database of Brazil [42]. We expected negative effects on detection from slope, as this variable would impose locomotion constraints to ground-dwelling birds, so that birds would walk less in steep slopes (reducing the probability of being photographed by our camera traps). We also would expect a negative relationship between detection probability and temperature and precipitation because many birds are less active during warmer and rainy days [27]. The wet season would increase detection for both species because this season coincides with their reproduction period when tinamous are generally more active [21].

### Data analysis

#### Time activity

We used the time of captures (converted to radians) to create a 24h activity pattern for brown and tataupa tinamou. We used kernel density estimation on circular data to characterize the activity patterns of each species (following [48, 49]). Then, we calculated the coefficient of overlap (Δ_1_) between the two species using the overlap package version 0.2.6 [48] in R software [50]. The coefficient of overlap ranges from 0 (no overlap) to 1 (complete overlap), and a low coefficient of overlap indicates temporal segregation [49]. Confidence intervals were obtained as percentile intervals from 10,000 bootstrap samples [48].

#### Single-species occupancy models

Occupancy modeling provides estimates of a site being occupied by the focal species while accounting for detection probability (*p*; [13]). We constructed the detection histories (H) of the tinamous for each site over ten consecutive week-long sampling occasions during each season. For each site and sampling occasion, species detection was recorded as “1” while non-detection was recorded as “0”. Even though tinamous have small home ranges, we recognize that movement could cause some sample units that are used by the species to be unoccupied at some sampling occasions, causing us to view occupancy as use of the sample unit by the focal species.

We used a three-step approach while modeling the occupancy of each tinamou using single-species multi-season models, as follows. 1) We determined the scale that best represents each species’ response to the habitat, using a general model for *p* (that contained as many potential covariates as possible, i.e., *p(*Season+Temp+Rain+Slope)) and allowed *Ψ* to vary (following [13]) by only the focal covariate measured at different buffer sizes (Table B in S1 File). By using a general model for the parameters that were not investigated within a specific model set, we reduced the possibility that imposed constraints (on *p*, for example) would result in residual sampling variation being “attributed” to variation in occupancy. When different spatial scales were equally plausible (i.e., difference of less than two AIC between them), we chose to use the scale that was closest to the species home range scale (i.e., approx. 20 ha) in the next step. 2) We investigated the influence of habitat-related variables on the occupancy probabilities (*Ψ*). We allowed *Ψ* to be constant (*Ψ*(.)), to vary by a single covariate or a combination of two covariates (additive effects), using each covariate at its scale of strongest response for each species (from the previous step) and a general model for *p* (Table C in S1 File). 3) We used the top-rank model for *Ψ* (from the previous step) and investigated which covariate(s) best explained the detection probability (*p*) (Table D in S1 File). We used the second and third step to narrow candidate covariates to be used in the co-occurrence models (similarly to [36]), as the latter may require large numbers of parameters and candidate models [17]. We treated the dry season as one season and the wet season as a second season. As we had only two seasons, and relatively small sample size, we were not able to investigate sources of variation in colonization and extinction. Therefore, we held colonization and extinction constant in all analyses.

We evaluated candidate models and estimated parameters using PRESENCE software [51], in which linear-logistic models were fit to determine the covariates that best explained the occupancy and detection probabilities. We ranked candidate models using Akaike’s Information Criterion (AIC; [52]) and considered the covariate(s) from the top-ranked model(s) (ΔAIC < 2) as the best determinant(s) of the species’ occupancy or detection. In addition, to assess the relative importance of each covariate, we summed the Akaike weights (*w*_*i*_) for the single-species models across all the models (*i*) where that covariate was present and examined the 95% confidence intervals (CIs) to see whether the β parameters describing the relationships overlapped 0 or not [52]. We applied model-averaging [52] to estimate overall occupancy of each species at our study area, using PRESENCE software [51].

#### Co-occurrence occupancy models

To investigate if the presence or detection of one species at a sampling site influences the presence or detection of the other species, we used two-species occupancy models [14]. In this step, we incorporated the top-ranked covariates from the single-species occupancy models, to account for habitat preferences and variables influencing their detection probability. We used the *Ψ*^Ba^/*r*^*Ba*^ parameterization in program PRESENCE [51], assuming that the brown tinamou (approx. 444 g) would be interspecifically dominance (e.g., exhibiting aggressive behavior) to tataupa tinamou (approx.220 g), given that bird size is positively correlated to interspecific dominance in birds [11, 12]. We initially tried to fit multi-season models, but could not obtain convergence. We thus used only data from the dry season (season with most records and which the single-species habitat modeling most closely applied) to estimate co-occurrence patterns. We estimated the following probabilities: *Ψ*^A^ (occupancy probability of the dominant species, i.e., the brown tinamou), *Ψ*^BA^ (occupancy probability of the subordinate species, i.e., the tataupa tinamou, when the dominant is present), and *Ψ*^Ba^ (occupancy probability of the subordinate species in the absence of the dominant species). We built a set of *a priori* models that assumed that the presence of the dominant species influenced the subordinate (*Ψ*^BA^ ≠ *Ψ*^Ba^), and constrained models where the occupancy of the subordinate was independent of presence of the dominant species (*Ψ*^BA^ = *Ψ*^Ba^). We incorporated the best covariates from single-species models, to account for habitat effects, and used a general model for *p*, that contained as many *p* parameters as possible, and the best detection covariates we identified from single-species models (see Table 1).

**Table 1:** Co-occurrence occupancy models used to evaluate the role of interspecific interactions and habitat partitioning between two sympatric ground-dwelling birds, the brown tinamou (*Crypturellus obsoletus*) and tataupa tinamou (*C*. *tataupa*), in a seasonal Atlantic forest remnant in Brazil. Models indicate the same (=) or different (≠) β parameters for the conditional *Ψ* probabilities. Models with ΔAIC < 2 are marked in bold. For detailed description of occupancy parameters see Methods section.

Occupancy Model ^†^	Occupancy Covariates ^‡^	ΔAIC	K	*w*_*i*_	LL
***Ψ***^**A**^ **≠ *Ψ***^**BA**^ **= *Ψ***^**Ba**^	**Elevation**^**A**^ **≠ Elevation**^**BA**^ **= Elevation**^**Ba**^	**0**	**12**	**0.62**	**558.34**
***Ψ***^**A**^ **≠ *Ψ***^**BA**^ **≠ *Ψ***^**Ba**^	**Elevation**^**A**^ **≠ Elevation**^**BA**^ **= Elevation**^**Ba**^	**1.83**	**13**	**0.25**	**558.17**
*Ψ*^A^ ≠ *Ψ*^BA^ ≠ *Ψ*^Ba^	Elevation^A^ ≠ Elevati006Fn^BA^ ≠ Elevation^Ba^	3.27	14	0.12	557.61
*Ψ*^A^ ≠ *Ψ*^BA^ = *Ψ*^Ba^	No covariate	10.59	10	0.00	572.93
*Ψ*^A^ ≠ *Ψ*^BA^ ≠ *Ψ*^Ba^	No covariate	12.41	11	0.00	572.75
*Ψ*^A^ = *Ψ*^BA^ = *Ψ*^Ba^	No covariate	15.24	9	0.00	579.58

K = no. of parameters. *w*_*i*_ = Akaike weight. LL = twice the negative log-likelihood.

^† ^All occupancy models included a general model for *p* (*p*^A^ *≠ r*^A^ *≠ p*^B^ *≠ r*^BA^ *≠ r*^Ba^; Slope^A^ ≠ Slope^B^; Temperature).

^‡ ^Covariates indicate whether the effect of elevation is the same or different for each occupancy probability.

For detection probability, we estimated the following parameters: *p*^A^ (probability of detecting the dominant species, given the absence of the subordinate), *p*^B^ (probability of detecting the subordinate, given the absence of the dominant), *r*^A^ (probability of detecting the dominant, given both are present), *r*^BA^ (probability of detecting the subordinate, given both are present and the dominant is detected), *r*^Ba^ (probability of detecting the subordinate species, given both are present and the dominant is not detected). We built a set of *a priori* models assuming that the detection probabilities of each species were independent of the presence or detection of the other (*p*^A^ *= r*^A^ and *p*^B^ *= r*^BA^ *= r*^Ba^), models where only the subordinate was influenced by the presence of the dominant (*p*^A^ *= r*^a^ and *p*^B^ *≠ r*^BA^ *= r*^Ba^) and models where each species was influenced by the presence and detection of the other (*p*^A^ *≠ r*^A^ and *p*^B^ *≠ r*^BA^ *≠ r*^Ba^). We incorporated the best covariates for detection probabilities from single-species models, keeping the same effects in this two-species co-occurrence modeling.

We also calculated the species interaction factor (SIF) for occupancy (*φ*, following [17]) and detection (δ, adapted from [17]) in order to address whether the two species are more or less likely to co-occur than expected by chance alone, as follows:

$$\phi =\frac{\Psi^{A}\Psi^{\mathrm{BA}}}{\Psi^{A}\left(\Psi^{A}\Psi^{\mathrm{BA}}+\left({1\text{-}\Psi }^{A}\right)\Psi^{\mathrm{Ba}}\right)}\;\delta =\frac{r^{A}r^{\mathrm{BA}}}{r^{A}\left(r^{A}r^{\mathrm{BA}}+\left({1\text{-}r}^{A}\right)\;r^{\mathrm{Ba}}\right)}$$ (1)

The SIF is a ratio of how likely the two species are to co-occur compared to what would be expected under a hypothesis of independence [17]. We performed model-averaging to obtain parameter estimates [52]. If SIF = 1, the species co-occur or are detected together about as frequently as expected under the null hypothesis of independence, while SIF < 1 or > 1 indicates respectively avoidance (co-occur or are detected less frequently than expected by chance) or aggregation (species are more likely to co-occur or to be detected than expected by chance).

We used AIC to rank candidate models. To infer about the co-occurence patterns, we considered the estimated parameters (*Ψ*^A^, *Ψ*^BA^, *Ψ*^Ba^, *r*^A^, *p*^A^, *p*^B^, *r*^Ba^, *r*^BA^) of the top-ranked model(s) (ΔAIC < 2), and the calculated SIF.

#### Ethics statement

Secretariat of Environment of the Jundiaí City Hall provided permission to conduct this project at the Biological Municipal Reserve (no permit number), and private owners provided permission through verbal authorization. During this research, the animals were observed in their natural environment and none of them were captured or handled. Therefore, there are no protocols to be reported to institutional or governmental agencies that regulate animal research.

## Results

### Time activity

**Fig 2:**
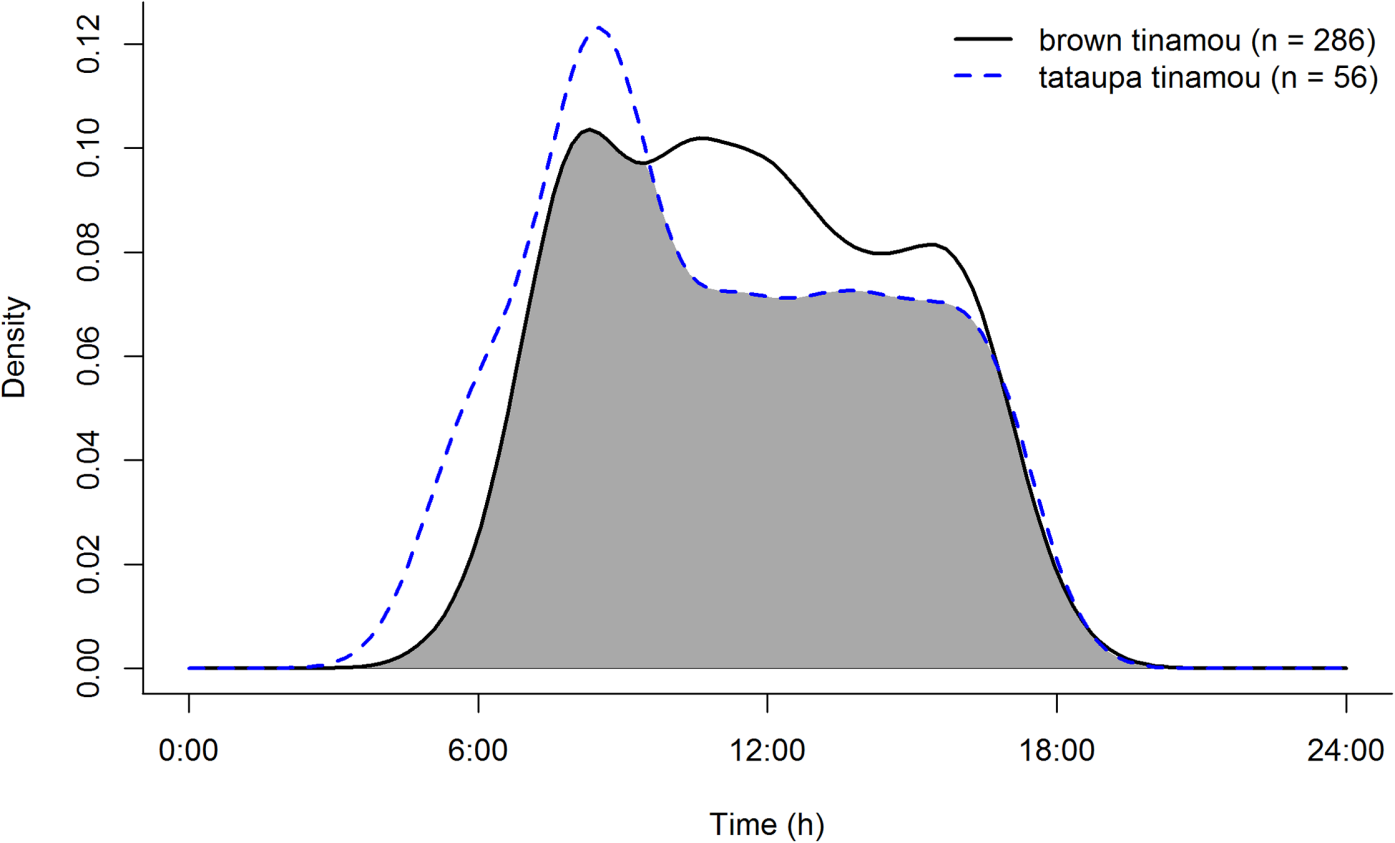
Time activity of two sympatric Neotropical tinamous, the brown tinamou (*Crypturellus obsoletus*) and tataupa tinamou (*C*. *tataupa*), in a continuous seasonal Atlantic forest in Brazil. Sample sizes in parentheses indicate the number of detections for each species. Gray shading indicates the overlap in species activity.

We had a total of 342 tinamous detections (N_brown tinamou_ = 286; N_tataupa tinamou_ = 56). Both species were strictly diurnal, with a slight peak of activity in early morning (Fig 2). We found evidence that the two tinamous overlap in activity to a great extent throughout the day (Δ_1_ = 0.88, 95% CI: 0.79–0.94), rejecting our hypothesis for time segregation.

### Single species occupancy models

#### Occupancy probability

**Fig 3:**
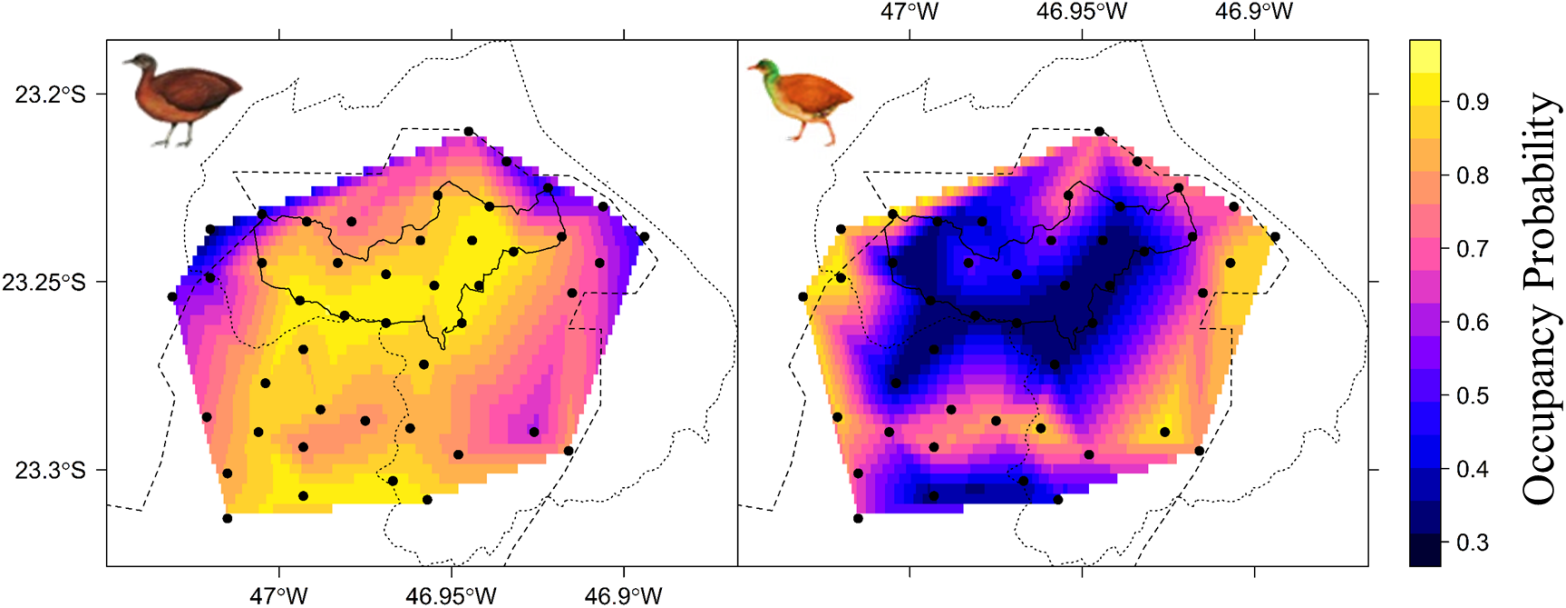
Estimated site occupancy probability of brown tinamou (*Crypturellus obsoletus*, left) and tataupa tinamou (*C*. *tataupa*, right) at an Atlantic forest remnant in Brazil based on camera trap survey data. Darker colors indicated lower occupancy probabilities. This map was obtained from interpolation of model-averaged site occupancy probabilities.

**Fig 4:**
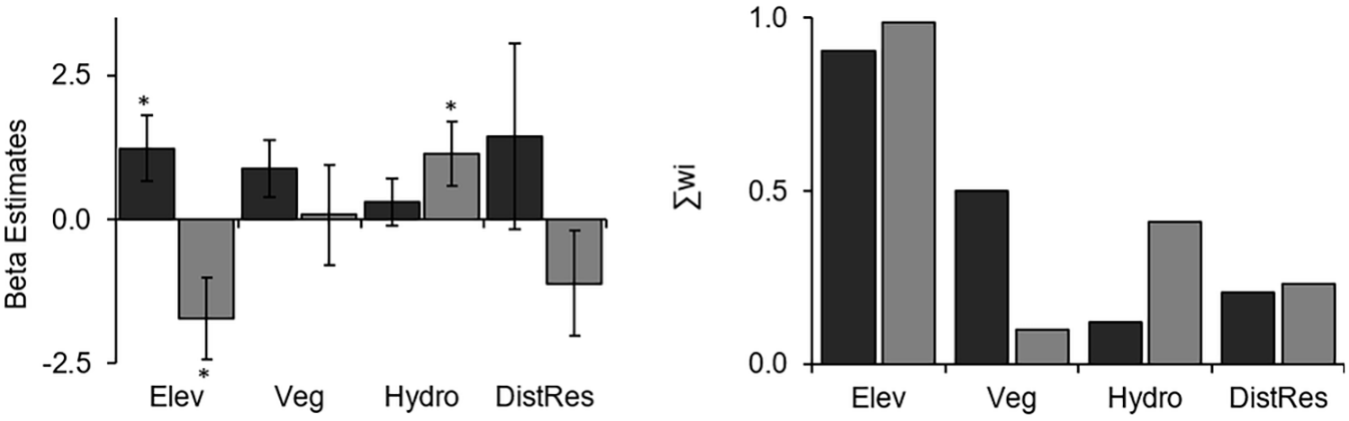
Influence of elevation (Elev), high-quality vegetation cover (Veg), hydrographic density (Hydro), and weighted distance to the Biological Municipal Reserve border (DistRes) in the occupancy probability of brown (*Crypturellus obsoletus*, black bars) and tataupa tinamou (*C*. *tataupa*, gray bars) in a large Atlantic forest remnant and relative importance (sum of Akaike weight, ∑*w*_*i*_) of each covariate. * indicates that approximate 95% confidence interval does not include zero.

Covariates measured at different spatial scales were not always similarly supported. For instance, high-quality vegetation measured within a 500-m radius buffer and hydrographic density at 1000-m for brown tinamou were better ranked than these same covariates measured at different spatial scales. For tataupa tinamou, elevation and hydrographic density at 200-m were more supported than at other spatial scales (Table B in S1 File). There was little support for the null model (*Ψ*(.)) for either species in the single-species models (ΔAIC > 7 for both model sets, Table C in S1 File). The species occupy different and opposite portions of the landscape (Fig 3). Elevation was the main predictor of their occupancy, with a significant positive effect for the brown tinamou, and a significant negative effect for the tataupa tinamou (Fig 4; Table C and Fig A in S1 File). We also had some support that high quality vegetation and hydrographic density may affect the occupancy of brown tinamou and tataupa tinamou, respectively, but to a minor degree (Fig 4). Thus, we selected only elevation to run the co-occurrence models.

#### Detection probability

While terrain slope, season, and temperature (to a minor degree) emerged as main predictors decreasing the detection of brown tinamou, tataupa tinamou detection was not highly influenced by any of the analyzed covariates (Table D and Fig B in S1 File). Because slope and temperature were among the top-ranked models for both species, we selected these covariates while running the co-occurrence models. We did not select season for the next step because we opted to use single season co-occurrence models (see Methodssection).

### Co-occurrence occupancy models

#### Occupancy probability

**Fig 5:**
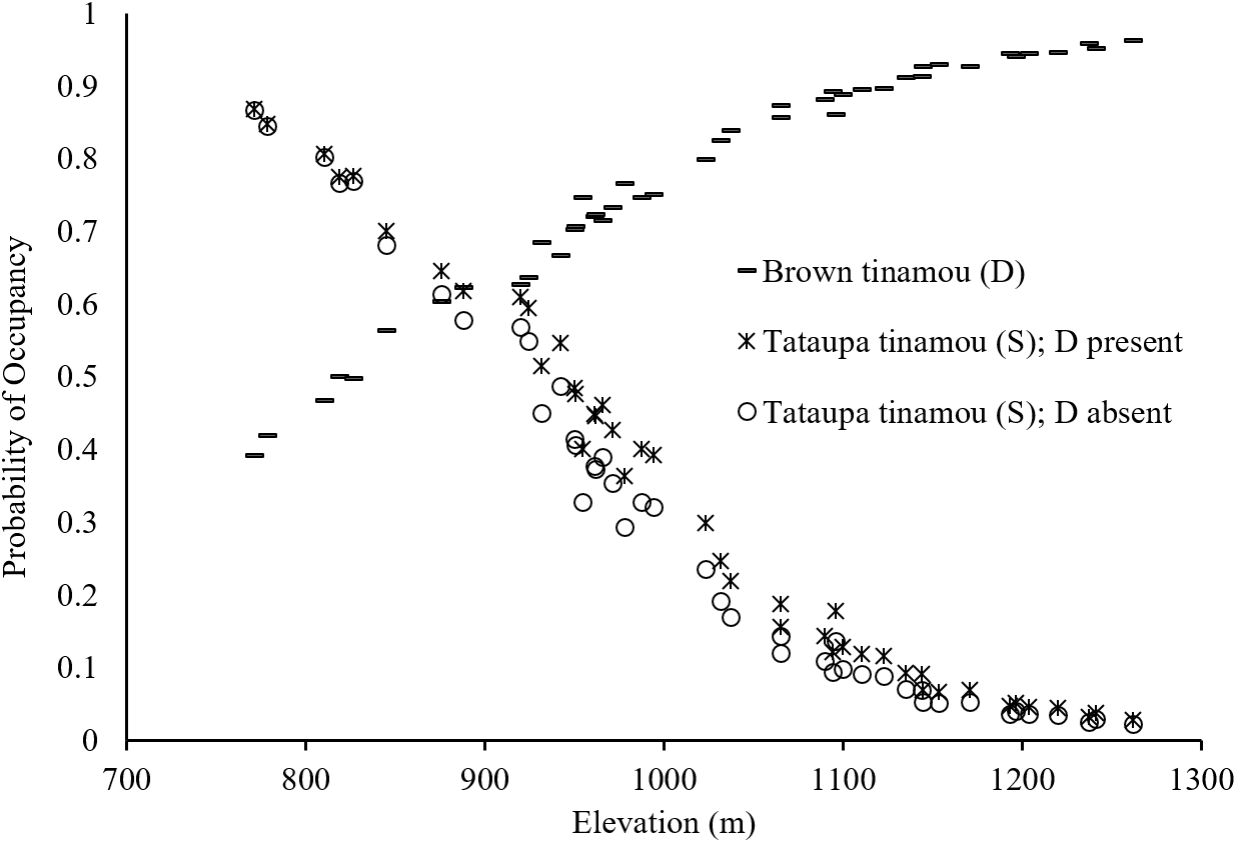
Occupancy probability estimates of brown tinamou (*Crypturellus obsoletus*) and tataupa tinamou (*C*. *tataupa*) according to the elevational gradient of Serra do Japi, Brazil. Plotted probabilities correspond to model-averaged estimates of occupancy for the brown tinamou (lines), which is the dominant species (D), and the tataupa tinamou (subordinate, S) in two different states: when D is present (crosses) or absent (circles).

We found no evidence that the presence of brown tinamou (dominant) influences the occupancy of tataupa tinamou (subordinate species) (i.e., *Ψ*^BA^ = *Ψ*^Ba^; Table 1). After accounting for elevation, the occupancy probability of tataupa tinamou varied little with the presence of brown tinamou (Fig 5; Table E in S1 File), even though the second ranked model suggested some influence. In addition, models in which we incorporated the effects of habitat covariates on occupancy were relatively better than models without covariates (∑*w*_*i*_ = 0.99 vs ∑*w*_*i*_ < 0.01). The species interaction factor also provided little evidence of dependence of the occupancy of one species on that of the other ($\hat{\varphi }$ = 0.96).

#### Detection probability

We had overall low support for one species affecting the detection probability of the other (i.e., more evidence that *p*^A^ *= r*^A^ or *p*^B^ *= r*^BA^ *= r*^Ba^; Table 2). The detection of the tataupa tinamou varied little with the presence or detection of the brown tinamou (Table E in S1 File). Furthermore, the species interaction factor indicated that detections were independent of the presence and/or detection of the other species ($\hat{\delta }$ = 0.99).

**Table 2:** Co-occurrence detection models used to evaluate the effect of detection and/or presence of one ground-dwelling tinamou species on the detection of the other (the brown tinamou (*Crypturellus obsoletus*) and tataupa tinamou (*C*. *tataupa*)) in a seasonal Atlantic forest remnant in Brazil.

Detection Model^†^	Detection Covariates ^‡^	ΔAIC	K	*w*_*i*_	LL
***p***^**A**^ ***= r***^**A**^ ***≠ p***^**B**^ ***= r***^**BA**^ ***= r***^**Ba**^	**Slope**^**A**^ **≠ Slope**^**B**^**,Temp**	**0**	**9**	**0.50**	**560.4**
*p*^A^ *= r*^a^ *≠ p*^B^ *≠ r*^BA^ *= r*^Ba^	Slope^A^ ≠ Slope^B^,Temp	2.00	10	0.18	560.4
*p*^A^ *= r*^A^ *≠ p*^B^ *= r*^BA^ *= r*^Ba^	No covariate	2.43	6	0.15	568.83
*p*^A^ *≠ r*^A^ *≠ p*^B^ *≠ r*^BA^ *≠ r*^Ba^	Slope^A^ ≠ Slope^B^,Temp	3.94	12	0.07	558.34
*p*^A^ *= r*^a^ *≠ p*^B^ *≠ r*^BA^ *= r*^Ba^	No covariate	4.43	7	0.05	568.83
*p*^A^ *≠ r*^A^ *≠ p*^B^ *≠ r*^BA^ *≠ r*^Ba^	No covariate	5.12	9	0.04	565.52
*p*^A^ *= r*^A^ *= p*^B^ *= r*^BA^ *= r*^Ba^	No covariate	18.58	5	0	586.98

Models indicate the same (=) or different (**≠**) β parameters for the conditional *p* or *r* probabilities. Models with ΔAIC < 2 are marked in bold. For detailed description of detection parameters see Methods section. K = no. of parameters. *w*_*i*_ = Akaike weight. LL = twice the negative log-likelihood. Slope = terrain slope. Temp = temperature.

^†^All detection models included the best model for occupancy from the co-occurrence models (*Ψ*^A^ ≠ *Ψ*^BA^ = *Ψ*^Ba^; Elevation^A^ ≠ Elevation ^BA^ = Elevation^Ba^.

^‡ ^Covariates indicate that the effect of terrain slope is different for each species (Slope^A^ ≠ Slope^B^), while temperature is the same.

## Discussion

We applied a co-occurrence model framework to two morphologically similar sympatric ground-dwelling bird species, while explicitly incorporating habitat characteristics and controlling for detection probability in a large Atlantic Forest remnant. We had no evidence for a dependent pattern of co-occurrence, as species occupied sites independently. Instead, occupancies appeared to result from differences in habitat preferences between the two tinamous species.

Similar sympatric species of many taxa rely on time partitioning and are active in different times of day to avoid interspecific competition [9, 53], including birds [7, 8, 9]. However, we had no evidence for time partitioning between the two ground-dwelling birds analyzed here because their time activity highly overlapped. Brown tinamou and tataupa tinamou had similar and homogeneous activity throughout the morning and the afternoon. This homogeneous daily activity also was found for other tinamous (little tinamou, *C*. *soui*, [54]; and black tinamou, *Tinamus osgoodi*, [35]), but two distinct peaks of activity throughout the day are also possible within this bird family (e.g., solitary tinamou, *T*. *solitarius*; [34, 36]). We suggest that more studies analyze tinamous’ time use in areas where they occur in sympatry to understand whether their distinct time activities are the result of time partitioning or if they are simply differences in species’ biology.

The occupancy of the two ground-dwelling birds had opposite relationships with elevation, supporting our first hypothesis, which predicted that habitat partitioning would be an important mechanism to allow their co-existence. After accounting for these habitat preferences, negative interspecific interactions (e.g., exclusion or avoidance) do not appear to influence the distribution of the two tinamous because the presence and/or detection of one tinamou did not affect the presence and/or detection of the other. These results highlight the importance of elevational gradients to segregate species (similarly to [14]). Interestingly, the larger species (brown tinamou) had higher occupancy probabilities at the upland than its smaller congener. Perhaps, the difference of approx. 3°C between the lowest and the highest portion of the elevational range in our study area (based on a decrease of 5°C per 1000 m of elevation; [55]) is selecting for a more tolerant species at the mountaintop, as larger birds are more likely to conserve heat and tolerate lower temperatures [56].

Even though habitat partitioning also applies to other sympatric and congener species in different taxa, including birds [57], mammals [58], and reptiles [59], species co-occurrence patterns seem to vary broadly depending on the role played by competitive interactions and/or habitat selection. For instance, while some animals may co-occur using time partitioning in combination with habitat selection and different foraging behavior (e.g., mice; [58]), in others, interspecific interactions among congeners and habitat segregation simultaneously influence species co-occurrence (e.g., passerines; [57]). In some cases, however, habitat partitioning alone seems to influence the co-occurrence of species (e.g., passerines; [60, 61]), such as the tinamous analyzed here. Perhaps when species spatial ecology is a direct function of habitat heterogeneity, habitat diversity can facilitate co-occurrence of species [4, 57]. In fact, habitat heterogeneity tends to have a positive effect on bird diversity [62] and favors patchy distributions [63]. Birds can exhibit fine-scale foraging behavior when food sources are heterogeneously distributed [64], and refine their preferences for certain vegetation types at a finer-scale [65], which could be the case of the birds studied here. On the other hand, habitat homogeneity may increase the occurrence and density of a stronger competitor for which that habitat is suitable, which in turn would decrease the subordinate species occurrence in that habitat, and their distribution would likely be more a function of competitive interactions [4]. Such could be the scenario in the absence of an elevational gradient (i.e., homogeneity), in which brown tinamou and tataupa tinamou could avoid or exclude each other. Moreover, interspecific interactions may also be playing a smaller role for our two tinamous because large protected forests, such as our study area, provide suitable habitat to maintain viable populations of sympatric species [66]. Then, habitat destruction (e.g., fragmentation) could limit space segregation and habitat partitioning between the two tinamous, and, therefore, strengthen interspecific competition [67].

We thus believe that there are two possible explanations for the tinamous habitat partitioning. Evolutionarily, habitat partitioning may be a result of segregation through historical species co-evolution to reduce interspecific competition and facilitate co-occurrence [68, 69, 70]. Alternatively, species may have evolved separately, become adapted to different sets of habitat, and started to co-exist regionally [68]. While it is difficult to discern which of these scenarios is true for our two tinamous, we provide evidence that tinamous may choose specific types of microhabitat, consistently with previous naturalistic research, even though these lacked rigorous methodology (e.g., [23, 24, 71, 72]). The fact that one tinamous affected neither the occupancy nor the detection of the other adds to this evidence.

Although competition alone is regarded as the leading driver of co-occurrence patterns in many species (see [5] for a systematic review), our results indicate that habitat characteristics can play a bigger role than direct interspecific interactions in regulating co-existence of some species. Our results corroborate findings in other closely related species, such as snakes [4], salamanders [14], and small carnivores [31]. By exploring habitat preferences and co-occurrence patterns simultaneously, we were able to define the effects of habitat and achieve more accurate estimation of species interactions while overcoming issues related to imperfect detection with occupancy models.

As a last comment, our procedure (camera traps and occupancy modeling) respected the nature of interaction between species while collecting data, and, therefore, could be used when surveying other cryptic and vocally shy animals, such as other ground-dwelling birds (e.g., other tinamous, ratites, and cracides species), felids, or ungulates.

## Supporting information

S1 File Figure A. Relationship between occupancy probability and elevation for two sympatric Neotropical tinamous, the brown tinamou (*Crypturellus obsoletus*) and tataupa tinamou (*C*. *tataupa*) in a continuous seasonal Atlantic Forest remnant Brazil. Figure B. Influence of the analyzed climate and habitat variables in the detection probabilities of two sympatric Neotropical tinamous, the brown tinamou *(Crypturellus obsoletus*) and tataupa tinamou (*C*. *tataupa*) in a large Atlantic Forest remnant in Brazil and relative importance of each variable. Table A. Spearman’s correlation matrix for the site covariates measured at different scales (buffer sizes) at a large Atlantic Forest remnant in Brazil. Table B. Model selection analysis for occupancy probability (*Ψ*) covariates (high-quality vegetation, hydrographic density, and elevation) measured at different scales (buffer sizes, from 200 m to 1000 m) for two sympatric Neotropical tinamous, the brown tinamou (*Crypturellus obsoletus*) and tataupa tinamou (*C*. *tataupa*), in a seasonal large Atlantic Forest remnant in Brazil. Table C. Single-species occupancy models used to evaluate the effects of geographic, environmental and protection status features on the occupancy probability (*Ψ*) of two sympatric Neotropical tinamous, the brown tinamou (*Crypturellus obsoletus*) and tataupa tinamou (*C*. *tataupa*), in a seasonal large Atlantic Forest remnant in Brazil. Table D. Single-species detection models used to evaluate the effects of sampling occasion covariates on the detection probability (*p*) of two sympatric Neotropical tinamous, the brown tinamou (*Crypturellus obsoletus*) and tataupa tinamou (*C*. *tataupa*), in a seasonal large Atlantic Forest remnant in Brazil. Table E. Co-occurrence model average estimates of occupancy (*Ψ*) and detection parameters (*p* and *r*) of two sympatric Neotropical tinamous, the brown tinamou (*Crypturellus obsoletus*) and tataupa tinamou (*C*. *tataupa*), in a seasonal large Atlantic Forest remnant in Brazil.
(DOCX)
