# Supplementary material for: When habitat matters: Habitat preferences can modulate co-occurrence patterns of similar sympatric species
Source: PLoS One. 2017 Jul 26;12(7):e0179489. doi: 10.1371/journal.pone.0179489 (PMC5528253; doi:10.1371/journal.pone.0179489)
Supplement: S1 File — Figure A. Relationship between occupancy probability and elevation for two sympatric Neotropical tinamous, the brown tinamou (Crypturellus obsoletus) and tataupa tinamou (C. tataupa) in a continuous seasonal Atlantic Forest remnant Brazil. Figure B. Influence of the analyzed climate and habitat variables in the detection probabilities of two sympatric Neotropical tinamous, the brown tinamou (Crypturellus obsoletus) and tataupa tinamou (C. tataupa) in a large Atlantic Forest remnant in Brazil and relative importance of each variable. Table A. Spearman’s correlation matrix for the site covariates measured at different scales (buffer sizes) at a large Atlantic Forest remnant in Brazil. Table B. Model selection analysis for occupancy probability (Ψ) covariates (high-quality vegetation, hydrographic density, and elevation) measured at different scales (buffer sizes, from 200 m to 1000 m) for two sympatric Neotropical tinamous, the brown tinamou (Crypturellus obsoletus) and tataupa tinamou (C. tataupa), in a seasonal large Atlantic Forest remnant in Brazil. Table C. Single-species occupancy models used to evaluate the effects of geographic, environmental and protection status features on the occupancy probability (Ψ) of two sympatric Neotropical tinamous, the brown tinamou (Crypturellus obsoletus) and tataupa tinamou (C. tataupa), in a seasonal large Atlantic Forest remnant in Brazil. Table D. Single-species detection models used to evaluate the effects of sampling occasion covariates on the detection probability (p) of two sympatric Neotropical tinamous, the brown tinamou (Crypturellus obsoletus) and tataupa tinamou (C. tataupa), in a seasonal large Atlantic Forest remnant in Brazil. Table E. Co-occurrence model average estimates of occupancy (Ψ) and detection parameters (p and r) of two sympatric Neotropical tinamous, the brown tinamou (Crypturellus obsoletus) and tataupa tinamou (C. tataupa), in a seasonal large Atlantic Forest remnant in Brazil. (DOCX) [file pone.0179489.s001.docx]

**When Habitat Matters: Habitat Preferences can Modulate Co-Occurrence Patterns of Similar Sympatric Species**

César A. Estevo^1*, #a^, Mariana B. Nagy-Reis^1,2^, James D. Nichols^1^

^1^ United States Geological Survey, Laurel, Maryland, United States of America

^2^ Department of Animal Biology, University of Campinas, Campinas, São Paulo, Brazil

^#a^ Current Address: Avenida Juscelino Kubitschek 901, Paulínia, São Paulo, Brazil.

^*^ Corresponding author

E-mail: cesar_estevo@hotmail.com (CAE)

All authors contributed equally to this work.

# **S1 Appendix**

## **Single- species and co-occurrence occupancy models**

**
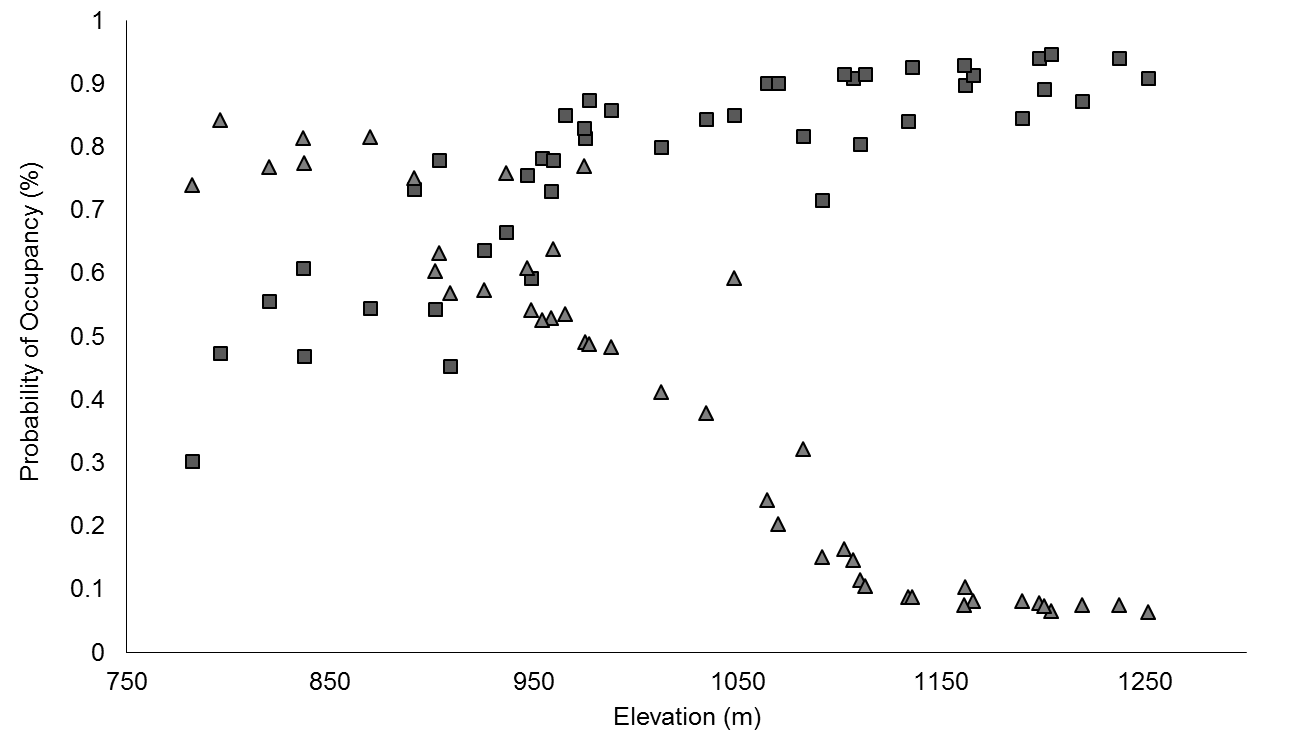
S1 Figure A. Relationship between occupancy probability and elevation for two sympatric Neotropical tinamous, the brown tinamou (*Crypturellus obsoletus*) and tataupa tinamou (*C. tataupa*) in a continuous seasonal Atlantic Forest remnant Brazil**. Filled squares = brown tinamou. Filled triangles = tataupa tinamou. Plotted probabilities correspond to single-species model-averaged estimates of occupancy.


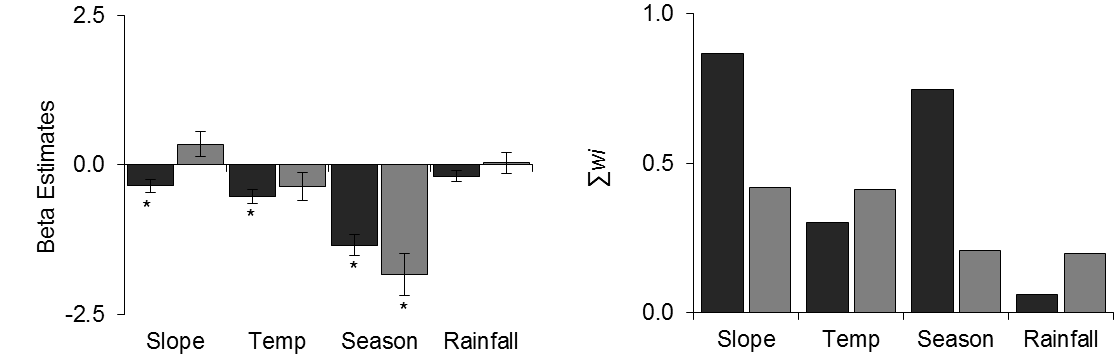


**S1 Figure B. Influence of the analyzed climate and habitat variables in the detection probabilities of two sympatric Neotropical tinamous, the brown tinamou *(Crypturellus* *obsoletus*) and tataupa tinamou (*C. tataupa*) in** **a large Atlantic Forest remnant in Brazil** **and relative importance of each variable.** Black bars = brown tinamou. Gray bars = tataupa tinamou. Slope = terrain slope. Temp = temperature. Season = rainy season. *∑w_i_ =* sum of Akaike weight. *** indicates that 95% confidence interval does not include zero*.*

**S1 Table A. Spearman’s correlation matrix for the site covariates measured at different scales (buffer sizes) at a large Atlantic Forest remnant in Brazil.**

|  | **Elev** | **Elev** | **Elev** | **Elev** | **Veget** | **Veget** | **Veget** | **Veget** |
| --- | --- | --- | --- | --- | --- | --- | --- | --- |
|  | **200** | **300** | **500** | **1000** | **200** | **300** | **500** | **1000*** |
| **Hydro200** | -0.16 | -0.11 | -0.05 | 0.04 | 0.21 | 0.24 | 0.26 | 0.26 |
| **Hydro300** | -0.21 | -0.17 | -0.09 | 0.00 | 0.15 | 0.19 | 0.21 | 0.19 |
| **Hydro500** | -0.16 | -0.13 | -0.07 | 0.03 | 0.20 | 0.23 | 0.23 | 0.18 |
| **Hydro1000** | -0.02 | 0.01 | 0.03 | 0.11 | 0.26 | 0.30 | 0.34 | 0.26 |
| **Elev200** | - | - | - | - | 0.07 | 0.14 | 0.24 | **0.52** |
| **Elev300** | - | - | - | - | 0.06 | 0.13 | 0.24 | **0.53** |
| **Elev500** | - | - | - | - | 0.05 | 0.13 | 0.25 | **0.54** |
| **Elev1000** | - | - | - | - | 0.08 | 0.16 | 0.27 | **0.58** |

The three landscape site covariates (Elev = elevation, Hydro = hydrographic density, Veget = percentage of high-quality forest cover) were measured at 200-, 300-, 500- and 1000 m spatial scales (buffer sizes). Highly correlated (r > 0.50) outcomes are in bold. * = excluded covariates.

**S1 Table B**. **Model selection analysis for occupancy probability (*Ψ*) covariates (high-quality vegetation, hydrographic density, and elevation) measured at different scales (buffer sizes, from 200 m to 1000 m) for two sympatric Neotropical tinamous, the brown tinamou (*Crypturellus obsoletus*) and tataupa tinamou (*C. tataupa*), in a seasonal large Atlantic Forest remnant in Brazil**.

| **Model** | **ΔAIC** | ***w_i_*** | **K** | **LL** |
| --- | --- | --- | --- | --- |
| **High-quality Vegetation** | | | | |
| **Brown tinamou** | |  |  |  |
| *Ψ*(vegetation1000) *p*(general) | 0 | 0.61 | 9 | 737.68 |
| *Ψ*(vegetation500) *p*(general) | 2.09 | 0.21 | 9 | 739.77 |
| *Ψ*(vegetation300) *p*(general) | 3.93 | 0.09 | 9 | 741.61 |
| *Ψ*(.) *p*(general) | 4.96 | 0.05 | 8 | 744.64 |
| *Ψ*(vegetation200) *p*(general) | 5.44 | 0.04 | 9 | 743.12 |
| **Tataupa tinamou** | |  |  |  |
| *Ψ*(.) *p*(general) | 0 | 0.34 | 8 | 279.76 |
| *Ψ*(vegetation200) *p*(general) | 1.00 | 0.20 | 9 | 278.76 |
| *Ψ*(vegetation300) *p*(general) | 1.24 | 0.18 | 9 | 279.00 |
| *Ψ*(vegetation500) *p*(general) | 1.64 | 0.15 | 9 | 279.40 |
| *Ψ*(vegetation1000) *p*(general) | 1.84 | 0.13 | 9 | 279.60 |
| **Hydrographic density** | | | | |
| **Brown tinamou** | |  |  |  |
| *Ψ*(.) *p*(general) | 0 | 0.38 | 8 | 744.64 |
| *Ψ*(hydro1000) *p*(general) | 1.64 | 0.17 | 9 | 744.28 |
| *Ψ*(hydro500) *p*(general) | 1.85 | 0.15 | 9 | 744.49 |
| *Ψ*(hydro300) *p*(general) | 1.88 | 0.15 | 9 | 744.52 |
| *Ψ*(hydro200) *p*(general) | 1.97 | 0.14 | 9 | 744.61 |
| **Tataupa tinamou** | |  |  |  |
| *Ψ*(hydro200) *p*(general) | 0 | 0.63 | 9 | 273.03 |
| *Ψ*(hydro300) *p*(general) | 2.27 | 0.20 | 9 | 275.30 |
| *Ψ*(hydro500) *p*(general) | 4.02 | 0.08 | 9 | 277.05 |
| *Ψ*(.) *p*(general) | 4.73 | 0.06 | 8 | 279.76 |
| *Ψ*(hydro1000) *p*(general) | 6.25 | 0.03 | 9 | 279.28 |
| **Elevation** | | | | |
| **Brown tinamou** | |  |  |  |
| *Ψ*(elevation300) *p*(general) | 0 | 0.27 | 9 | 736.24 |
| *Ψ*(elevation500) *p*(general) | 0.13 | 0.25 | 9 | 736.37 |
| *Ψ*(elevation1000) *p*(general) | 0.13 | 0.25 | 9 | 736.37 |
| *Ψ*(elevation200) *p*(general) | 0.45 | 0.22 | 9 | 736.69 |
| *Ψ*(.) *p*(general) | 6.40 | 0.01 | 8 | 744.64 |
| **Tataupa tinamou** | |  |  |  |
| *Ψ*(elevation300) *p*(general) | 0 | 0.37 | 9 | 265.38 |
| *Ψ*(elevation200) *p*(general) | 0.05 | 0.36 | 9 | 265.43 |
| *Ψ*(elevation500) *p*(general) | 0.88 | 0.24 | 9 | 266.26 |
| *Ψ*(elevation1000) *p*(general) | 4.37 | 0.04 | 9 | 269.75 |
| *Ψ*(.) *p*(general) | 12.38 | 0.00 | 8 | 279.76 |

High-quality vegetation at 1000 m was correlated with other variables (r > 0.50), so we did not consider it for further occupancy analysis. K = no. of parameters. *w_i_* = Akaike weight. LL = twice the negative log-likelihood. *Ψ =* occupancy probability. *p =* detection probability. Hydro = hydrographic density. Vegetation = high-quality vegetation. (.) = null model. general = Season + Temperature + Rainfall + Terrain Slope.

**S1 Table C. Single-species occupancy models used to evaluate the effects of geographic, environmental and protection status features on the occupancy probability (*Ψ*) of two sympatric Neotropical tinamous, the brown tinamou (*Crypturellus obsoletus*) and tataupa tinamou (*C. tataupa*), in a seasonal large Atlantic Forest remnant in Brazil**.

| **Model** | **ΔAIC** | ***w_i_*** | **K** | **LL** | **Beta Estimates (Standard Error)** | | | | |
| --- | --- | --- | --- | --- | --- | --- | --- | --- | --- |
|  |  |  |  |  | Elevation | Vegetation | Hydrography | Reserve | |
| **Brown tinamou** |  |  |  |  |  |  |  |  |  |
| ***Ψ*(elevation200 + vegetation500 ) *p*(general)** | 0 | 0.42 | 10 | 733.17 | 1.23 (0.57)* | 0.88 (0.49) |  |  |  |
| ***Ψ*(elevation200) *p*(general)** | 1.52 | 0.20 | 9 | 736.69 | 1.56 (0.78)* |  |  |  |  |
| ***Ψ*(elevation200 + reserve) *p*(general)** | 1.65 | 0.19 | 10 | 734.82 | 1.88 (0.99) |  |  | 1.43 (1.61) |  |
| *Ψ*(elevation200 + hydrography1000 ) *p*(general) | 2.95 | 0.10 | 10 | 736.12 | 1.31 (0.54)* |  | 0.30 (0.41) |  |  |
| *Ψ*(vegetation500) *p*(general) | 4.60 | 0.04 | 9 | 739.77 |  | 0.80 (0.38)* |  |  |  |
| *Ψ*(vegetation500 + hydrography1000) *p*(general) | 6.50 | 0.02 | 10 | 739.67 |  | 0.85 (0.42)* | 0.14 (0.43) |  |  |
| *Ψ*(vegetation500 + reserve) *p*(general) | 6.56 | 0.02 | 10 | 739.73 |  | 0.78 (0.38)* |  | 0.09 (0.43) |  |
| *Ψ*(.) *p*(general) | 7.47 | 0.01 | 8 | 744.64 |  |  |  |  |  |
| *Ψ*(hydrography1000) *p*(general) | 9.11 | 0.00 | 9 | 744.28 |  |  | 0.22 (0.37) |  |  |
| *Ψ*(reserve) *p*(general) | 9.30 | 0.00 | 9 | 744.47 |  |  |  | 0.16 (0.39) |  |
| *Ψ*(hydrography1000 + reserve) *p*(general) | 11.02 | 0.00 | 10 | 744.19 |  |  | 0.20 (0.38) | 0.12 (0.40) |  |
| **Tataupa tinamou** |  |  |  |  |  |  |  |  |  |
| ***Ψ*(elevation200 + hydrography200) *p*(general)** | 0 | 0.40 | 10 | 262.61 | 1.72 (0.71)* |  | 1.14 (0.56)* |  |  |
| ***Ψ*(elevation200) *p*(general)** | 0.82 | 0.26 | 9 | 265.43 | 4.23 (2.48) |  |  |  |  |
| ***Ψ*(reserve + elevation200) *p*(general)** | 1.12 | 0.23 | 10 | 263.73 | 5.48 (2.69)* |  |  | 1.11 (0.91) |  |
| *Ψ*(elevation200 + vegetation200) *p*(general) | 2.82 | 0.10 | 10 | 265.43 | 4.18 (2.60)* | 0.08 (0.87) |  |  |  |
| *Ψ*(hydrography200) *p*(general) | 8.42 | 0.01 | 9 | 273.03 |  |  | 1.12 (0.50)* |  |  |
| *Ψ*(hydrography200 + reserve) *p*(general) | 9.72 | 0.00 | 10 | 272.33 |  |  | 1.14 (0.50)* | 0.35 (0.43) |  |
| *Ψ*(hydrography200 + vegetation200) *p*(general) | 9.81 | 0.00 | 10 | 272.42 |  | 0.36 (0.48) | 1.13 (0.51)* |  |  |
| *Ψ*(.) *p*(general) | 13.15 | 0.00 | 8 | 279.76 |  |  |  |  |  |
| *Ψ*(vegetation200) *p*(general) | 14.15 | 0.00 | 9 | 278.76 |  | 0.41 (0.43) |  |  |  |
| *Ψ*(reserve) *p*(general) | 14.74 | 0.00 | 9 | 279.35 |  |  |  | 0.25 (0.39) |  |
| *Ψ*(reserve + vegetation200) *p*(general) | 15.88 | 0.00 | 10 | 278.49 |  | 0.39 (0.44) |  | 0.21 (0.40) |  |

Models with ΔAIC < 2 are marked in bold. + indicates additive effect of covariates.*** indicates that 95% confidence interval does not include zero. K = no. of parameters. *w_i_* = Akaike weight. LL = twice the negative log-likelihood*. p =* detection probability. Hydro = hydrographic density. Vegetation = high-quality vegetation. Reserve = weighted distance to reserve border. (.) = null model. general = Season + Temperature + Rainfall + Terrain Slope.

**S1 Table D**. **Single-species detection models used to evaluate the effects of sampling occasion covariates on the detection probability (*p*) of two sympatric Neotropical tinamous, the brown tinamou (*Crypturellus obsoletus*) and tataupa tinamou (*C. tataupa*), in a seasonal large Atlantic Forest remnant in Brazil**.

| **Model** | **ΔAIC** | ***w_i_*** | **K** | **LL** | **Beta Estimates (Standard Error)** | | | |
| --- | --- | --- | --- | --- | --- | --- | --- | --- |
|  |  |  |  |  | Season | Temp | Rain | Slope |
| **Brown tinamou** |  |  |  |  |  |  |  |  |
| ***Ψ*(elevation200 + vegetation500) *p*(season + slope)** | 0 | 0.67 | 8 | 740.49 | -1.35 (0.18)* |  |  | -0.35 (0.11)* |
| *Ψ*(elevation200 + vegetation500) *p*(temp + slope) | 2.43 | 0.20 | 8 | 742.92 |  | -0.53 (0.11)* |  | -0.30 (0.11)* |
| *Ψ*(elevation200 + vegetation500) *p*(temp + season) | 5.29 | 0.05 | 8 | 745.78 | -1.10 (0.19)* | -0.32 (0.15)* |  |  |
| *Ψ*(elevation200 + vegetation500) *p*(temp + rain) | 5.52 | 0.04 | 8 | 746.01 |  | -0.49 (0.11)* | -0.19 (0.10)* |  |
| *Ψ*(elevation200 + vegetation500) *p*(season + rain) | 7.15 | 0.02 | 8 | 747.64 | -1.21 (0.18)* |  | -0.17 (0.10)* |  |
| *Ψ*(elevation200 + vegetation500) *p*(temp) | 7.66 | 0.01 | 7 | 750.15 |  | -0.51 (0.11)* |  |  |
| *Ψ*(elevation200 + vegetation500) *p*(season) | 8.24 | 0.01 | 7 | 750.73 | -1.25 (0.17)* |  |  |  |
| *Ψ*(elevation200 + vegetation500) *p*(slope + rain) | 19.33 | 0.00 | 8 | 759.82 |  |  | -0.25 (0.10)* | -0.27 (0.11)* |
| *Ψ*(elevation200 + vegetation500) *p*(rain) | 23.34 | 0.00 | 7 | 765.83 |  |  | -0.24 (0.10)* |  |
| *Ψ*(elevation200 + vegetation500) *p*(slope) | 24.51 | 0.00 | 7 | 767.00 |  |  |  | -0.26 (0.11)* |
| *Ψ*(elevation200 + vegetation500) *p*(.) | 28.13 | 0.00 | 6 | 772.62 |  |  |  |  |
| **Tataupa tinamou** |  |  |  |  |  |  |  |  |
| ***Ψ*(elevation200 + hydro200) *p*(.)** | 0 | 0.18 | 6 | 266.77 |  |  |  |  |
| ***Ψ*(elevation200 + hydro200) *p*(slope + temp)** | 0.09 | 0.17 | 8 | 262.86 |  | -0.36 (0.23) |  | 0.34 (0.21) |
| ***Ψ*(elevation200 + hydro200) *p*(slope)** | 0.49 | 0.14 | 7 | 265.26 |  |  |  | 0.26 (0.21) |
| ***Ψ*(elevation200 + hydro200) *p*(temp)** | 0.55 | 0.14 | 7 | 265.32 |  | -0.29 (0.24) |  |  |
| ***Ψ*(elevation200 + hydro200) *p*(season)** | 1.89 | 0.07 | 7 | 266.66 | -1.84 (0.35)* |  |  |  |
| ***Ψ*(elevation200 + hydro200) *p*(rain)** | 1.97 | 0.07 | 7 | 266.74 |  |  | 0.03 (0.17) |  |
| *Ψ*(elevation200 + hydro200) *p*(slope + season) | 2.21 | 0.06 | 8 | 264.98 | -1.86 (0.35)* |  |  | 0.28 (0.21) |
| *Ψ*(elevation200 + hydro200) *p*(rain + temp) | 2.39 | 0.05 | 8 | 265.16 |  | -0.31 (0.24) | 0.07 (0.18) |  |
| *Ψ*(elevation200 + hydro200) *p*(season + temp) | 2.46 | 0.05 | 8 | 265.23 | -1.73 (0.37)* | -0.33 (0.27) |  |  |
| *Ψ*(elevation200 + hydro200) *p*(slope + rain) | 2.48 | 0.05 | 8 | 265.25 |  |  |  | 0.26 (0.21) |
| *Ψ*(elevation200 + hydro200) *p*(season + rain) | 3.81 | 0.03 | 8 | 266.58 |  |  | 0.02 (0.18) |  |

Models with ΔAIC < 2 are marked in bold. + indicates additive effect of covariates. *** indicates that 95% confidence interval does not include zero. K = no. of parameters. *w_i_* = Akaike weight. LL = twice the negative log-likelihood. *Ψ =* occupancy probability. Hydro = hydrographic density. Vegetation = high-quality vegetation. Season = rainy season. Rain = rainfall. (.) = null model.

**S1 Table E**. **Co-occurrence model average estimates of occupancy (*Ψ*) and detection parameters (*p* and *r*) of two sympatric Neotropical tinamous, the brown tinamou (*Crypturellus obsoletus*) and tataupa tinamou (*C. tataupa*), in a seasonal large Atlantic Forest remnant in Brazil**.

| Parameter | Model Averaged Estimate |
| --- | --- |
| *Ψ*^A^ | 0.77 |
| *Ψ*^BA^ | 0.34 |
| *Ψ*^Ba^ | 0.30 |
| *p^A^* | 0.42 |
| *p^B^* | 0.17 |
| *r^A^* | 0.40 |
| *r^BA^* | 0.17 |
| *r^Ba^* | 0.17 |

# *Ψ*^A^ = occupancy probability of the dominant species (i.e., the brown tinamou). *Ψ*^BA^ = occupancy probability of the subordinate species (i.e., the tataupa tinamou), when the dominant is present. *Ψ*^Ba^ = occupancy probability of the subordinate species in the absence of the dominant species. *p*^A^ = probability of detecting the dominant species, given the absence of the subordinate. *p*^B^ = probability of detecting the subordinate, given the absence of the dominant. *r*^A^ = probability of detecting the dominant, given both are present. *r*^BA^ = probability of detecting the subordinate, given both are present and the dominant is detected. *r*^Ba^ = probability of detecting the subordinate species, given both are present and the dominant is not detected.
